# Matching on health status to estimate the effects of outpatient care and social factors in patients with COPD: a Norwegian registry‑based study

**DOI:** 10.1186/s12913-026-14285-9

**Published:** 2026-03-03

**Authors:** Tron Anders Moger, Jon Helgheim Holte, Olav Amundsen, Silje Bjørnsen Haavaag, Anne Edvardsen, Line Kildal Bragstad, Ragnhild Hellesø, Nina Køpke Vøllestad, Trond Tjerbo

**Affiliations:** 1 Department of Health Management and Health Economics, Institute of Health and Society, University of Oslo, Oslo, Norway; 2 Department of Public Health Science and Interdisciplinary Science, Institute of Health and Society, University of Oslo, Oslo, Norway; 3 Department of Pulmonary Medicine, Akershus University Hospital, Lørenskog, Norway; 4 Department of Rehabilitation Science and Health Technology, Oslo Metropolitan University, Oslo, Norway

**Keywords:** COPD, Hospital admissions, Readmissions, Outpatient care, Registry data, Matching techniques

## Abstract

Isolating the effects of outpatient care on hospitalisation in observational data is challenging. We estimate the effects of factors believed to influence hospital admissions in patients with COPD. The factors were related to outpatient care and social factors, motivated by recommendations in COPD guidelines and the literature. We applied linked national and municipal registry data on 25,369 COPD patients in Oslo and Trondheim 2008–2019. Yearly frequency and type of healthcare contacts (respiratory and non-respiratory, across providers), together with comorbidities, were used as proxies for patients’ health status. We constructed samples matched on the full set of proxy variables to estimate the effect of each factor on future COPD hospital admissions. Overall, 9.8% of follow-up years had at least one COPD hospital admission. Findings indicated beneficial effects of several of the factors. Care interaction between general practitioner (GP) and other health providers, follow-up by GP within 30 days after COPD hospital discharge and counselling/psychotherapy by GP showed significant reductions of 2.9, 4.9 and 1.4% points in the rate of next-year COPD hospital admissions, respectively. The effects are noteworthy when extrapolated to the entire Norwegian COPD population. A reduction of 1% point could result in up to 1,500 fewer patients experiencing one or more COPD hospital admissions during a year. In the absence of detailed clinical information, creating samples that are multidimensionally matched on health service use and comorbidities as pragmatic proxies for health status could be a viable approach for estimating the effects of other factors.

**Supplementary Information:**

The online version contains supplementary material available at 10.1186/s12913-026-14285-9.

## Introduction

Chronic obstructive pulmonary disease (COPD) represents a progressive respiratory condition marked by persistent symptoms and restricted airflow that can lead to structural airway damage over time [1]. As the third leading cause of death globally, COPD accounted for over 3 million deaths and approximately 390 million cases in 2019 [2, 3]. The disease burden is projected to grow due to ongoing exposure to risk factors and global population aging [1, 2, 4].

A central goal in COPD management involves preventing exacerbations—acute episodes of symptom deterioration that often require hospitalisation [5]. Hospitalisation represents the most significant direct healthcare cost for COPD patients [6, 7] and substantially impacts quality of life. While clinical and sociodemographic factors associated with COPD admission risk have been extensively studied [7–11], less is known about how outpatient care coordination, quality and social support influence admission risk outside clinical trial settings. COPD patients navigate a complex web of primary, specialist, and long-term care providers, with multimorbidity being frequent [5, 12], making care coordination and support potentially important for outcomes. However, findings may be limited by sample size and follow-up time [13–15]. Due to the chronic, slowly progressing nature of COPD, periods with worsening of the condition and high use of health care services may be followed by periods with relative stability, both of which may be influenced by care coordination, quality and support.

The potential of registry data for research on chronic diseases has been highlighted recently [16–19]. In Norway, individual level linkage across primary, specialist and long term care registries enables large, population based studies with long follow up, reducing problems of small sample size and limited observation time. However, registry studies face challenges from their observational nature and from the frequent lack of clinical measures of disease severity in administrative data. In a previous population-based analysis of COPD patients identified in administrative registries, we found that prior healthcare use, particularly combinations of contacts across multiple providers, was strongly associated with next year COPD hospital admissions after adjustment for comorbidities and sociodemographic variables [20]. We also explored potential indicators of more COPD focused care (e.g. provider interaction, spirometry in GP offices, continuity of care, timely post-discharge follow-up, and early rehabilitation), but those analyses were exploratory, used the full sample, and may have been biased because patients without such interventions could include many who were simply too healthy to require them.

The present paper aims to estimate effects of outpatient and social factors on COPD hospitalisation using linked Norwegian registry data. To address missing clinical severity and build on our prior work, we match patients on yearly healthcare contacts (frequency and provider type for respiratory and non‑respiratory diagnoses) and comorbidities, treating these measures as pragmatic proxies for health status at the time of matching. The assumption is that matching on these multidimensional proxies captures overall health status, allowing estimation of the effects of GP follow-up, timeliness and type of rehabilitation, psychosocial support by GPs, family support, education and immigrant status. Unmatched control observations are assumed to be too healthy for the factor of interest to be relevant and are thus excluded from the analysis. By further adjusting for sociodemographic and socioeconomic variables that are indirectly related to health, we estimate the effects of the independent variables mentioned above.

## Methods

### Sample selection

Based on a previous analysis of these data [20], we included patients residing in Oslo or Trondheim who were aged ≥ 40 at their first recorded contact with a primary COPD diagnosis (ICD‑10: J43–J44; ICPC‑2: R95) during 2009–2018. Patients were identified through linkage of the Norwegian Patient Registry (NPR, encompassing public inpatient and outpatient treatment and private rehabilitation services) and the Control and Payment of Reimbursement to Health Service Providers (KUHR, covering consultations with general practitioners, contract specialists, and physiotherapists). A minimum one-year follow-up period from the initial healthcare contact with COPD as the primary diagnosis was required for inclusion. The dataset encompasses all variables for both 2008 and 2019, enabling assessment of patient health status upon entry to the sample in 2009 and capturing of subsequent-year hospital admissions for patients entering 2018.

Additional data linkages were performed with the Regular General Practitioner registry (RGP, containing GP and practice characteristics), municipal electronic patient journals (MEPJ, documenting services from safety alarms to home nursing and institutional rehabilitation), socioeconomic and demographic data from Statistics Norway (SSB), and mortality data from the Cause of Death registry. Further details regarding this dataset are available in previous publications [20, 21]. This resulted in a sample of 25,369 individuals.

### Data structuring

From the sample selection, some patients have had the condition for several years when entering the sample, while others are incident cases. However, we have information on healthcare use and comorbidities in the year prior to first registered COPD diagnosis and assume that this adequately captures the COPD and overall health status of the patients when they enter the sample. To capture variation in health care use over time within patients, the data were organised per year of follow-up, giving repeated observations for each individual.

**Fig. 1:**
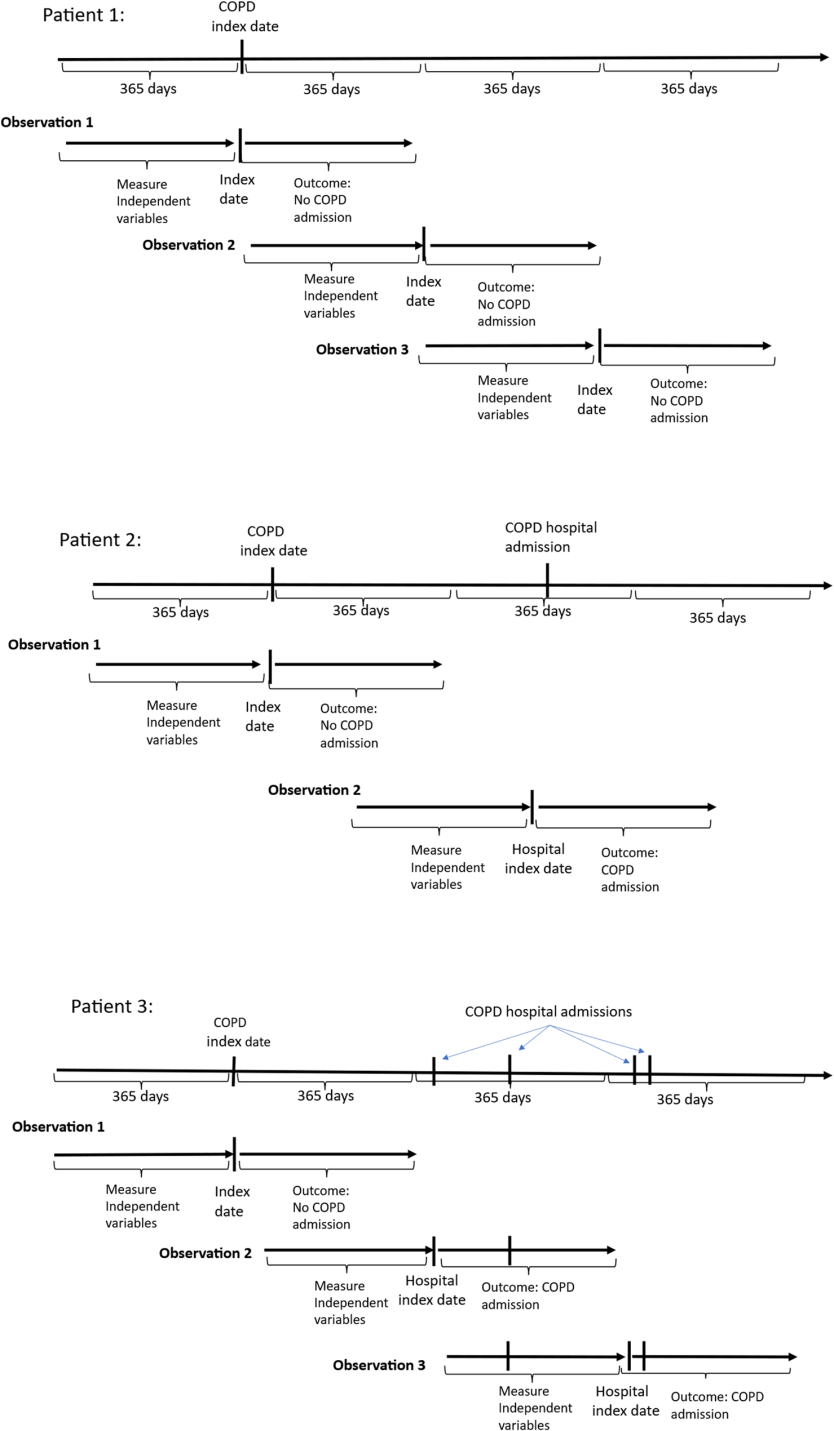
Construction of yearly observations from three example patients with data for four full years. Each yearly observation comprises two periods: a follow-up year during which COPD admissions are registered (the dependent variable), and a prior year during which independent variables are recorded (Tables 1 and 2). Patient 1 has no COPD admissions during the entire follow-up. Patient 2 has a single COPD admission. For this patient, two separate observations were constructed to avoid overlap between follow-up years. Patient 3 has multiple COPD admissions, and three separate observations were constructed for this patient, two of which are defined by COPD admission dates

For patients without any COPD hospital admissions during follow-up, the first follow-up year begins on the date of their first registered healthcare contact with a COPD diagnosis between 2009 and 2018. This date can occur at an arbitrary time in the patient’s life with the disease and lasts for one year, thus marking the start of the second follow-up year. The process continues until the final complete follow-up year, as illustrated for patient 1 in Fig. 1. In contrast, patients with COPD admissions during follow-up have their follow-up years first identified by the hospital admission dates. This means we capture healthcare use during the year immediately preceding the first admission in that follow-up year. Follow-up years without COPD admissions were further defined with no overlap within the same patient. For example, patient 2 in Fig. 1 has only one COPD admission during follow-up, which establishes a follow-up year. Given this admission, only one additional observation can be constructed for the patient. Patient 3 has multiple COPD admissions. Here it is possible to define two follow-up years with COPD admissions, and one follow-up year without admissions. Consequently, one observation for patient 3 also includes a COPD admission during the prior year.

**Table 1:** List of primary independent variables. NPR=Norwegian Patient Registry, KUHR=Control and Payment of Reimbursement to Health Service Providers, RGPR=Regular General Practitioner registry, MEPJ=municipality electronic patient journal

Variable	Definition	Source
**Outpatient follow-up by GP**		
Spirometry at GP	Indicator of spirometry performed at least once at GP or contract specialist in prior year	KUHR
Care interaction, respiratory diagnoses	Indicator of fees for interaction between either GP/specialist, GP/long-term care or GP/physiotherapist at least once in prior year	KUHR
Early follow-up by GP, respiratory diagnoses	Indicator of contact with GP within 30 days after at least one COPD hospital admission vs. none or only later follow-up in prior year	NPR/KUHR
Continuity of GP care	Indicator of continuity of care index for GP contact concentration above 0.6 in prior year (Scale range 0–1, higher values imply contact with fewer unique GPs, thus better continuity).	KUHR
**Rehabilitation**		
Early rehabilitation, respiratory diagnoses	Indicator of rehabilitation within 30 days following at least one COPD hospital admission vs. later rehabilitation (up to 6 months) after COPD hospital admissions in prior year	NPR/MEPJ
Supervised physical training	Indicator of procedure code for supervised and instructed physical training related to motor skills and/or fitness in public hospital in prior year	NPR
Rehabilitation private vs. public	Indicator of private or public (in-hospital, ICD-10 code Z50) rehabilitation in prior year	NPR
Rehabilitation in municipality vs. somatic	Indicator of rehabilitation from the municipality (both in institution and at home) or at public hospital (both in- and outpatient rehabilitation) in prior year	NPR/MEPJ
**Family support**		
Married/widowed vs. not married	Indicator of being married/widowed (thus either having a spouse or children) vs. not married	Statistics Norway
**Socioeconomic status**		
Education college/university vs. lower	Indicator of college/university education	Statistics Norway
First-generation immigrant	Indicator of being first-generation immigrant	Statistics Norway
**Care for vulnerable groups**		
Counselling and psychotherapy	Indicator of fees for counselling or psychotherapy with the patient and/or next-of-kin at GP in prior year	KUHR

The intuition behind the data structuring is as follows. Even if a patient had no COPD admissions during follow-up, there could be several providers (GP, specialist, outpatient hospital) contributing to the COPD care during some periods, thus indicating poor COPD health status. It is possible that the care provided in these periods contributed to avoiding hospitalisations. Similarly, if a patient had one or more COPD admissions in a prior year, outpatient care could still be provided during that year helping to avoid COPD admissions in the following year. The choice of using a yearly resolution in the data is partly pragmatic. The prior period must be sufficiently long to capture the health status of the patient, and the factors we analysed were either provided few times per year (if at all) or may be preventive in the long-term. Observations were included up to the last complete follow‑up year. If the final follow‑up year was incomplete but contained a COPD hospital admission, that partial year was retained and classified as an observation with a COPD admission.

### Dependent variable

Hospital admissions were combined if discharge and admission dates were less than a day apart to capture transfers within and between hospitals. COPD hospital admissions were defined by ICD-10 codes J43, J44 as the main diagnosis. Using this definition, admissions were presumed to capture COPD exacerbations [22, 23].

### Independent variables

The independent variables were split into primary variables, which were assumed to directly or indirectly influence quality of care (Table 1), and secondary variables, which capture patients’ health status and were used for matching and risk adjustment (Table 2). The rationale for each variable in Table 1 can be summarised as follows, with definitions given in the table.

**Table 2:** List of secondary independent variables used for matching and risk adjustment in the analyses. NPR=Norwegian Patient Registry, KUHR=Control and Payment of Reimbursement to Health Service Providers, RGPR=Regular General Practitioner registry, MEPJ=municipality electronic patient journal

Variable	Definition	Source
**Matching variables for respiratory health status in prior year**		
Contact with GP, respiratory diagnoses	Number of contacts with respiratory diagnoses: 0, 1–2, 3–4, and 5 or more	KUHR
Emergency room contacts, respiratory diagnoses	Number of contacts with municipal emergency room: 1 if at least one, 0 otherwise	KUHR
Outpatient contact with hospital, respiratory diagnoses	Number of contacts: 1 if at least one, 0 otherwise	NPR
Hospital admissions, COPD	Number of admissions: 1 if at least one, 0 otherwise	NPR
**Matching variables for non-respiratory health status in prior year**		
Contact with GP, other diagnoses	Number of contacts with non-respiratory diagnoses: 0–2, 3–7, 8–14 and 15 or more	KUHR
Hospital admissions, non-COPD	Number of admissions: 1 if at least one, 0 otherwise	NPR
Comorbidities	Sum of indicators for each of 17 categories of comorbidities commonly associated with COPD, see Supplemental Material, Table S1 for details: 0, 1–2 and 3 or more	NPR/KUHR
Reception of long-term care services	Yearly indicator on reception of any long-term care services from the municipality, 1 if yes, 0 otherwise	MEPJ
**Sociodemographics and -economics used for risk adjustment**		
Updated yearly	Gross personal income, age	Statistics Norway
Constant	Gender, indicator of permanent disability pension prior to or during follow-up	Statistics Norway

Norwegian COPD guidelines recommend annual GP check-ups with spirometry [24]. Coordinated care for COPD patients has received attention in the literature, but with uncertain effects [13–15]. In KUHR, care interactions between GPs and other healthcare providers during consultations are recorded under specific fee codes, although not capturing the content of those interactions. Following COPD hospitalisations, guidelines stipulate GP follow-up within four weeks for cases without respiratory failure, with hospital-based follow-up recommended for more severe cases [24]. Earlier trials have demonstrated associations between primary care continuity and reduced hospitalisation rates [25, 26], with several studies specifically examining this relationship in COPD populations [27–29]. We used the Bice-Boxerman index [30] to measure continuity of care with the GP. The index ranges from 0 to 1, with higher values indicating greater concentration of visits with a single provider.

If in need of rehabilitation, the guidelines state that this should be initiated shortly (exact timing not specified) after discharge [24]. For consistency with the timing of GP follow-up, we compared patients receiving rehabilitation within four weeks vs. later. There is limited information in the data regarding the content of rehabilitation, thus it is challenging to study effects of specific types. However, the data included procedure codes for supervised physical training in public hospitals, enabling us to compare patients with supervised training during rehabilitation to those without. Further, in Norway there are private centers who offer rehabilitation on agreement with the public health enterprise. There is no extra cost for the patient. From a policy perspective, it is interesting to examine whether these achieved better outcomes than rehabilitation performed at a public hospital. Similarly, we compared risk of next-year hospital admissions depending on whether any rehabilitation during the year was provided by a public hospital or by the municipality (either in institution or at home).

Family support by having a spouse or children is associated with better COPD outcomes and health [31–33], and thus may reduce the likelihood of hospitalisations. Family support was defined as being married/widowed vs. unmarried when entering the sample, as both former categories should have higher likelihood of access to family support; less than 1% of the sample were registered as cohabitants. We also included two socioeconomic status variables, assumed related to health literacy. Having higher education or being a native Norwegian speaker could improve access to various types of healthcare, and both factors could influence the risk of future hospital admissions [34, 35]. Finally, a significant subgroup of COPD patients is particularly vulnerable, suffering from various lifestyle and mental disorders [36–38]. We defined vulnerable patients as having either alcoholism, mental disorders, depression or being on permanent disability pension. Short, problem-focused counselling and basic psychosocial support may be given by GPs in Norway. Receiving counselling or psychotherapy from a GP, either directly with the patient or involving the patient’s next of kin, could have a positive effect on the outcome compared to not receiving such care within this subgroup of patients [10, 11, 39].

When analysing each primary independent variable, observations were matched on the variables listed in Table 2, which were selected because they showed the strongest associations with next‑year COPD hospital admissions in a previous analysis [20]. Separating healthcare contacts in respiratory and non-respiratory care was based on the main diagnosis codes (ICD-10 in specialist care and ICPC in primary care). The sociodemographic and -economic variables listed in Table 2 were assumed indirectly associated with health and healthcare use and were included as additional risk adjusters when analysing the matched samples.

### Statistical methods

Descriptive statistics for the full sample are given as means and standard deviations for the continuous variables, and percentages for the categorical variables. We present statistics both per patient and per observation (where relevant). We used coarsened exact matching [40] to construct matched samples for each primary independent variable in Table 1, with the matching variables coarsened into categories to enable exact multidimensional matching. GP contacts with respiratory diagnoses were categorised into 0, 1–2, 3–4, and 5 or more in the prior year, while contacts with non-respiratory diagnoses were categorised into 0–2, 3–7, 8–14, and 15 or more contacts. We separated 0 from 1 to 2 respiratory contacts to distinguish no contact from likely routine follow-up and grouped higher counts to likely reflect progressively worse health status (some vs. many contacts). The cut‑offs for non‑respiratory contacts were set at the sample quartiles in the data. Emergency‑room visits and inpatient or outpatient hospital contacts in the prior year were coded as binary indicators (none vs. 1 or more), this also applied to receipt of any municipal long-term care (yes vs. no). Because these services were uncommon in the sample, binary indicators were considered sufficient. Comorbidities are relatively frequent in COPD patients [1], and were categorised as 0, 1–2, and 3 or more.

The degree of balance in matching variables before and after matching was measured by the multivariate L1 distance between observations scoring/not scoring on each primary independent variable. We report these for the raw data and for each matched dataset in the Supplemental Material, Table S2. In addition, we report the number of observations that were dropped due to the lack of matching observations. The number of dropped observations scoring on each primary independent variable should constitute only a small percentage of the corresponding total in the raw data. Otherwise, the observations may no longer be representative of the general population, making it challenging to interpret the estimated effects. Dropping control observations is less problematic, assuming these represent years where a patient is too healthy for the factor to be relevant.

Logistic regression models were used to analyse the effect of each primary independent variable on next-year COPD hospital admission (yes vs. no) in the matched datasets, adjusting for the sociodemographic and -economic covariates listed in Table 2. Because observations were constructed annually, patients could appear in multiple matched strata as their time‑varying exposures and service use changed over years. A previous analysis shows that within‑patient variation in healthcare use is greater than between‑patient variation [20], reflecting the episodic flares in COPD patients. Accordingly, standard errors clustered by id-number of the matching stratum were used. Further, as the number of observations varied across strata, they were weighted to ensure that the relative frequency of observations scoring/not scoring on the factor in each stratum was equal to that of the total matched sample [40]. To verify that the matched samples were similar in overall health, we compared the survival rates using Kaplan-Meier plots for both the matched samples and the raw data. The time scale was measured in years from the point of matching for the included observations and was split based on whether each factor was present or not. Some examples are provided in the Supplemental Material, Figures S1. Even though the matching aims to balance samples based on health status in a single year and is not directly related to long-term survival, the plots provide a rough illustration of the improvement in health status balance compared to the raw data. The data were analysed in Stata Version 17.0.

## Results

Descriptive statistics for the sample are shown in Table 3. In total, the raw data included 141,770 observations (follow-up years), from 25,369 patients. The COPD patients were elderly, with a fairly high percentage on disability pension, suffering from multimorbidity and receiving services from the municipality. The results per patient show that it was quite common to have many contacts with GP or using the different services in at least one year during the follow-up period, but less common as a proportion of all follow-up years. The frequency of the care services per patient in the list of primary independent variables varied from the common (60% had spirometry at GP at least once during follow-up) to the rare (4.4% had any rehabilitation following COPD hospital admissions in prior year). This likely reflects variation in use according to the severity of the COPD, some care is only relevant in the more severe cases.

**Table 3:** Descriptive statistics for the full sample. The per patient statistics are calculated using the maximum value during follow-up for gross personal income and the variables related to healthcare

Variable:	Per patient	Per observation
**Basic descriptives**		
Number of observations	25,369	141,770
Age, mean (SD)	65.7 (11.9)	
Years of follow-up, mean (SD)	5.5 (3.1)	
Gender, % female	52.6%	
Gross personal income, mean (SD)	466k (780k)	
Permanent disability pension prior to or during follow-up	34.9%	
Dead during follow-up	30.9%	
Average age at death	77.0 (10.4)	
**Outcome**: COPD hospital admissions in follow-up year	31.0%	9.8%
**Matching variables for respiratory health status in prior year**		
Contact with GP: 0	5.6%	32.1%
1–2	19.0%	26.2%
3–4	20.1%	16.0%
5 or more	55.3%	25.7%
Emergency room contacts	32.1%	10.6%
Outpatient contact with hospital	40.4%	17.6%
COPD hospital admissions	13.2%	2.8%
**Matching variables for non-respiratory health status in prior year**		
Contact with GP: 0–2	7.3%	23.0%
3–7	18.1%	28.8%
8–14	28.4%	24.7%
15 or more	46.2%	23.5%
Hospital admissions	66.9%	25.3%
Comorbidities: 0	5.0%	19.3%
1–2	42.6%	55.6%
3 or more	52.6%	25.1%
Receive long-term care services from the municipality	48.7%	20.8%
**Primary independent variables**		
**Outpatient follow-up**		
Spirometry at GP	60.1%	21.0%
Care interaction	29.1%	8.2%
Follow-up by GP within 30 days after at least one COPD hospital admissions in prior year	5.4%	1.2%
Only later/no follow-up by GP	7.8%	1.6%
Continuity of GP care above 0.6	92.4%	71.4%
**Rehabilitation follow-up**		
Any rehabilitation after COPD admission in prior year:	4.4%	1.5%
Within 30 days after at least one COPD admission in prior year	2.4%	0.5%
Later than 30 days	2.0%	0.4%
**Rehabilitation type and setting**		
Supervised physical training in public hospital	12.1%	2.8%
Rehabilitation in: Public hospital	10.4%	2.3%
Private center	9.0%	2.3%
Municipality	17.0%	4.2%
**Family support**		
Married/widowed	55.7%	
**Socioeconomic status**		
Education: College/university	18.9%	
First-generation immigrant	11.2%	
**Care for vulnerable groups**		
Counselling and psychotherapy	26.7%	8.2%

Table 4 shows the results of the matched analyses of the primary independent variables. The multidimensional balance for the variables used in the matching, and number of observations included or dropped in the matched samples for each primary independent variable, are given in Supplemental Material, Table S2. Several of the factors on outpatient follow-up showed a reduction in the COPD hospital admission rate if the factor was present compared to not. Although the marginal effects may seem modest, given the overall hospitalisation rate of 9.8% (Table 3), the magnitude of the effects were relatively large. Interestingly, being a first-generation immigrant was associated with a reduced risk, hence they do not seem to be a vulnerable group for COPD exacerbations. However, patients with primary/secondary education had higher admission rates than patients with college/university education. Hence, if one assumes that the health status in the two groups is identical in the matched sample, there is an effect of higher education. The same was found for being married/widower compared to not being married.

**Table 4:** Marginal effect and 95% confidence interval (CI) for each primary independent variable on COPD hospital admissions. Separate coarsened exact matched samples are used in the analysis of each factor, matching on the health variables in Table 2. Further adjustment is done by logistic regression on the matched samples, using the sociodemographic and -economic variables in Table 2

Variable:		Marginal effect (95% CI)	Hospitalisation rate with factor	Hospitalisation rate w/o factor
**Outpatient follow-up**				
Spirometry at GP		-0.4% (-0.8%, -0.0%)*	9.1%	9.5%
Care interaction		-2.9% (-4.5%, -1.3%)*	27.4%	30.3%
Follow-up by GP within 30 days after at least one COPD hospital admissions in prior year (vs. later/no GP follow-up)		-4.9% (-8.9%, -1.0%)*	44.5%	49.4%
Continuity of GP care above 0.6		-1.5% (-1.8%, -1.1%)*	8.9%	10.4%
**Rehabilitation follow-up**				
Rehabilitation within 30 days after at least one COPD admission in prior year (vs. only later rehabilitation)		-3.7% (-9.6%, 2.2%)	37.4%	41.1%
**Rehabilitation type and setting**				
Supervised physical training		-2.1% (-4.6, 0.4%)	15.0%	17.1%
Rehabilitation private vs. public hospital		-0.5% (-2.3%, 1.3%)	16.3%	16.8%
Rehabilitation in municipality vs. public hospital		-1.9% (-4.3%. 0.5%)	18.1%	20.0%
**Family support**				
Married/widower vs. not married		-0.7% (-1.1%, -0.1%)*	9.7%	10.4%
**Socioeconomic status**				
Education college/university vs. lower		-1.6% (-1.2%, -2.0%)*	6.5%	8.1%
First-generation immigrant vs. not		-1.5% (-1.9%, -1.0%)*	6.0%	7.5%
**Care for vulnerable groups**				
Counselling/psychotherapy vs. not		-1.4% (-2.4%, -0.3%)*	14.0%	15.4%

* significant at 5%-level

Care interaction fees, GP follow-up and rehabilitation after hospital admissions in the prior year, and physical training seemed provided during periods of worsening COPD or among severe or frail cases, as seen from the higher hospitalisation rates than the overall average of 9.8%. Some of the samples are relatively small, as they are conditional on COPD hospital admissions, rehabilitation or being in a vulnerable group (see Supplemental Material, Table S2).

For some independent variables, a relatively large proportion of observations with the factor of interest were discarded in the matching. This particularly applied to the comparisons between rehabilitation within 30 days vs. later and rehabilitation by the municipality vs. in a somatic hospital. As a result, the matched samples may no longer be representative. However, both analyses show that the survival curves exhibit similar mortality within the first year after matching, and comparable long-term trends in the matched and raw samples for observations where early or municipal rehabilitation is received (see Supplemental Material, Figure S1). This suggests limited selection bias towards only the healthiest or least healthy patients in the groups, which supports the validity of the results. Discarded observations with later or somatic rehabilitation come from patients using fewer healthcare services, hence the lower survival rates in the matched samples for these groups. This finding aligns with the assumption that they are in better health and thus not comparable.

## Discussion

### Main findings

We are not aware of any previous attempts to estimate marginal effects of factors that could influence the quality of outpatient follow-up for COPD patients using population-based observational data. With the caveat that we used a pragmatic approach to capture COPD severity and general health status, the results did show slight effects for most of the primary independent variables on COPD hospital admissions. There seemed to be risk reductions related to better outpatient care, such as timely follow-up and continuity by GP and the municipality, and interaction between the GP and other providers. Contacts where counselling or psychotherapy were provided also seemed to reduce the admission rate. In addition, there were no indications that first-generation immigrants are at a higher risk for hospital admissions. However, there were effects associated with higher education and marital status. The latter two may respectively be linked to better access to relevant outpatient services and informal care.

### Implications of findings

Establishing the potential effects of outpatient care and other factors that could impact the follow-up of COPD has important implications for both patients and hospital resource use. This is especially true for chronic diseases, where most of the follow-up occurs outside of the hospital. However, limited information on the exact content of outpatient care in administrative data, combined with the multimorbid, chronic nature of COPD and the observational nature of the data, makes this particularly challenging. Although the effects of the factors studied here seem small in relative terms, they may add up to significant reductions in the number of hospital admissions. Within the sample, a reduction of 1% point in the hospital admission rate would imply ca. 250 fewer patients with at least one admission per year, and twice that number in case of a 2% points reduction. In Norway, the prevalence of COPD among individuals over 40 years old is estimated to be 6–7% of a population of approximately 2.5 million. If these estimates are extrapolated to the entire population, a reduction of 1–2% points would correspond to approximately 1,500 to 3,000 fewer patients experiencing at least one admission per year. Therefore, the cumulative impact across the entire country could be substantial. For the primary independent variables which are defined conditional on previous COPD admissions and/or rehabilitation the effects are correspondingly much smaller, however. Although individual outpatient services are typically less costly than new hospitalisations, the overall cost impact is unclear because scaling up outpatient capacity has its own resource implications.

### Comparison to other studies

Several factors similar to those studied here have been evaluated in earlier trials, showing associations in the same direction for COPD hospital admissions and readmissions. This includes protective effects of self-management and rehabilitation on exacerbations [41–43], continuity of care by GP [27–29], increased health literacy both measured in interviews and by higher education [34, 35], and family network as measured by marital status [31, 33]. Marital status, physical activity, and factors related to being in a disadvantaged state, such as alcoholism, anxiety, and depression, have been shown to be linked to readmission rates [10, 11, 39]. This strengthens the findings of our study; however, the fact that these studies were conducted in different countries and use relative effect measures makes further comparison difficult. Furthermore, the conclusions regarding the variables included in the previous analysis [20] are also similar, even though a basic regression approach was applied to the full dataset in that paper. The only exception is the effect of rehabilitation within 30 days vs. later. While having similar effects in both analyses, the effect was not significant in the analysis presented here. This could be related to the smaller sample size due to the matching approach applied in this paper.

### Methodological considerations

When very large registry datasets are available, matching can be a viable strategy for identifying effects, as these datasets are likely to contain sufficient exact matches. An additional advantage is that coarsened exact matching automatically aims to achieve multidimensional balance across matching variables, rather than just unidimensional balance as is common with alternative methods. This is particularly important when matching variables are used as proxies for complex, unobserved factors such as COPD severity and its interaction with comorbid diagnoses. While this approach involves categorising some continuous or discrete variables, this is not necessarily problematic provided that measuring the variable within an interval offers sufficient information. Observations discarded due to the lack of exact matches may not be relevant for estimating the effect of an intervention. For example, the registration of interaction fees requires several providers to have been involved in the patient’s follow-up during the year and should be restricted to the more severe cases. Even so, conventional matching seems to be underused in the literature. Searching PubMed yields 482 hits for “coarsened exact matching” vs. 33,987 hits for “propensity score matching” and 7,898 hits for “inverse probability” and “weighting” (search performed Aug 10, 2025). Still, further analyses indicated that using inverse probability weighting or propensity score matching on our data might not substantially alter the conclusions, even if achieving only univariable balance on the matching variables. E.g. using inverse probability weighting and propensity score matching with default settings in Stata yielded 2.4% and 1.4% points reduction in the next-year COPD hospitalisation rate for interaction fees, respectively (both statistically significant). However, we believe that the methodological approach presented here could be an attractive alternative in settings with large amounts of data in general. For instance, for diagnoses that are acute and curable, with less complex care patterns and multimorbidity than in the case of COPD.

### Strengths and limitations

The main strength is the breadth of healthcare data that has been linked, enabling comprehensive information on the healthcare use and health status of the individuals in the sample. Identifying patients from register data also means that both newly diagnosed and patients living with COPD for a long time are included, as well as both mild and severe cases.

The main limitation is the assumption that healthcare use and comorbidities over the course of a year adequately capture the COPD and overall health status of the patient, which we cannot formally verify. Additionally, the high proportion of discarded observations in some analyses makes drawing firm conclusions difficult. Hence, the results should be interpreted with some caution. A simplistic way to check the validity of the assumption was to compare survival in the matched samples. However, since matching was based on healthcare use in a single year, achieving identical survival curves for the two groups over several years is somewhat unrealistic, as patients’ health may vary considerably over time.

A further limitation comes from relying on primary care diagnoses both as inclusion criteria and for classifying comorbidities. Ideally, diagnostic codes with pulmonary function tests, laboratory results, or imaging should have been available to confirm the COPD diagnosis. Since primary care visits typically address multiple health issues simultaneously, the selection of which diagnosis gets recorded in the data may be somewhat arbitrary, particularly regarding the timing of initial COPD diagnosis, as multimorbidity is common in the patients. This is why we categorised contacts broadly by respiratory and non-respiratory diagnosis in the analysis, rather than COPD and other diagnoses. Information on smoking status is also not available. The fact that data from only two municipalities were included may reduce generalisability, as access to care related to travel distance and few services in rural parts cannot be considered. On the other hand, heterogeneity related to these factors is reduced.

## Conclusions

With the caveat that we used health‑service characteristics as pragmatic proxies for health status in COPD patients, several factors thought to influence follow‑up were associated with a reduced likelihood of hospital admission. The findings further showed that although the effects seem small, the consequences may be substantial. A similar approach as used in this study could be relevant for other diagnoses as well.

## Supplementary Information

Below is the link to the electronic supplementary material.

Supplementary Material 1

## Data Availability

The datasets used in the current study are based on national registries and are not publicly available. Access to pseudonymised data from the national registries are only granted through application to the Norwegian Centre for Research Data and Regional Committees for Medical and Health Research Ethics.

## Abbreviations

COPD: Chronic obstructive pulmonary disease
GP: General Practitioner
ICD-10: International classification of diseases, 10th version
ICPC-2: International Classification of Primary Care, 2nd Edition
KUHR: Control and Payment of Reimbursement to Health Service Providers
MEPJ: Municipal electronic patient journals
NPR: Norwegian patient registry
RGP: Regular General Practitioner registry
SSB: Statistics Norway
