# Supplementary material for: Matching on health status to estimate the effects of outpatient care and social factors in patients with COPD: a Norwegian registry‑based study
Source: BMC Health Serv Res. 2026 Mar 3;26:478. doi: 10.1186/s12913-026-14285-9 (PMC13064038; doi:10.1186/s12913-026-14285-9)

**Supplemental Material**

**Table S1:** Definitions of comorbidities by ICD-10 and ICPC-2 codes.

| **Variable:** | **ICD-10:** | **ICPC-2:** | **Sample prevalence** |
| --- | --- | --- | --- |
| Alcoholism | F10 | P15-P16 | 10% |
| Anemia | D5*-D6* | N80-N82 | 14% |
| Cancer (except lung cancer) | All C except C3 | A79, B72-B74, D74-D77, F74, H75, K72, L71, N74, R84-R85, S77, T71, U75-U77, X75-X77, Y77-Y78 | 24% |
| Cardiovascular disease | I25, I48, I70-79 | K76, K78-80, K92 | 41% |
| Dementia | F00-F03, G30 | P70 | 6% |
| Depression and anxiety | F32-F34, F40-F41 | P74, P76 | 23% |
| Diabetes | E10-E14 | T89-T90 | 17% |
| Heart failure | I50 | K77 | 18% |
| Hypertension | I10-I15 | K86-K87 | 46% |
| Kidney disease or failure | N18-19 | U88, U99 | 10% |
| Lung cancer | C3* | R84-R85 | 5% |
| Mental disorders | F20-F31 | P72-P73, P75, P77, P79-P80, P82, P98-P99 | 8% |
| Myocardial infarction (incl. angina pectoris) | I20-I22 | K74-K75 | 19% |
| Obesity | E65-E66 | T82 | 5% |
| Osteoporosis | M81 | L95 | 15% |
| Stroke | I60-I64, G45 | K89-K91 | 14% |
| Underweight | E40-E46 | T05, T08, T11, T28, T91, T99 | 31% |

**Table S2:** Summary statistics for the coarsened exact matching samples used to analyse each primary independent variable. Multivariate balance (L1) across matching variables in the full data (141,770 observations), multivariate balance and number of observations in the matched sample. Perfect global balance is indicated by L1 = 0, and larger values indicate larger imbalance between observations receiving/not receiving the care. A maximum value of L1 = 1 indicates complete separation.

| **Variable:** | **L1 raw data** | **L1 matched sample** | **No. matched observations**  **(w/wo factor)** | **No. non-matched obs. w/wo factor** |
| --- | --- | --- | --- | --- |
| **COPD-related follow-up** |  |  |  |  |
| Spirometry at GP | 0.35 | 9.7*10^-14^ | 32,756/105,874 | 66/3,074 |
| Care interaction | 0.52 | 8.4*10^-14^ | 11,477/122,678 | 189/7,426 |
| Follow-up by GP within 30 days after at least one COPD hospital admissions in prior year (vs. later/no GP follow-up)* | 0.53 | 5.3*10^-16^ | 616/951 | 133/1,259 |
| Continuity of GP care above 0.6 | 0.19 | 3.9*10^-14^ | 98,852/39,938 | 2,387/593 |
| **Rehabilitation follow-up** |  |  |  |  |
| Rehabilitation within 30 days after at least one COPD hospital admission in prior year (vs. later rehabilitation)** | 0.49 | 2.7*10^-16^ | 385/341 | 323/207 |
| **Rehabilitation type and setting** |  |  |  |  |
| Supervised physical training*** | 0.50 | 5.3*10^-14^ | 1,025/1,652 | 135/475 |
| Rehabilitation private vs. public**** | 0.40 | 1.2*10^-15^ | 2,457/2,525 | 546/491 |
| Rehabilitation in municipality vs. somatic***** | 0.71 | 2.3*10^-15^ | 2,792/1,856 | 2,764/1,044 |
| **Informal care** |  |  |  |  |
| Married/widower vs. not married | 0.30 | 3.0*10^-14^ | 76,877/61,686 | 1,870/1,337 |
| **Socioeconomic status** |  |  |  |  |
| Education college/university vs. lower | 0.13 | 1.2*10^-13^ | 26,893/112,384 | 49/2,444 |
| First-generation immigrant vs. not | 0.28 | 5.2*10^-15^ | 16,252/117,475 | 89/7,954 |
| **Care for vulnerable groups** |  |  |  |  |
| Counselling/psychotherapy vs. not****** | 0.36 | 5.3*10^-14^ | 7,824/51,017 | 26/2,239 |

*Maximum no. of observations is 2,959 (observations with COPD hospital admissions in prior year)

** Maximum no. of observations is 1,256 (observations with any rehabilitation following COPD hospital admissions in prior year)

*** Maximum no. of observations 3,287 (observations with rehabilitation in public hospital in prior year)

**** Maximum no. of observations 6,019 (observations with rehabilitation in private center or public hospital in prior year)

***** Maximum no. of observations 8,456 (observations with rehabilitation in municipal or public hospital in prior year)

****** Maximum no. of observations 61,106 (patients with either permanent disability pension, alcoholism, depression or mental disorders)

**Figures S1:** Examples of survival in matched samples vs. raw data for some of the primary independent variables. Analysis time=years since time of matching.


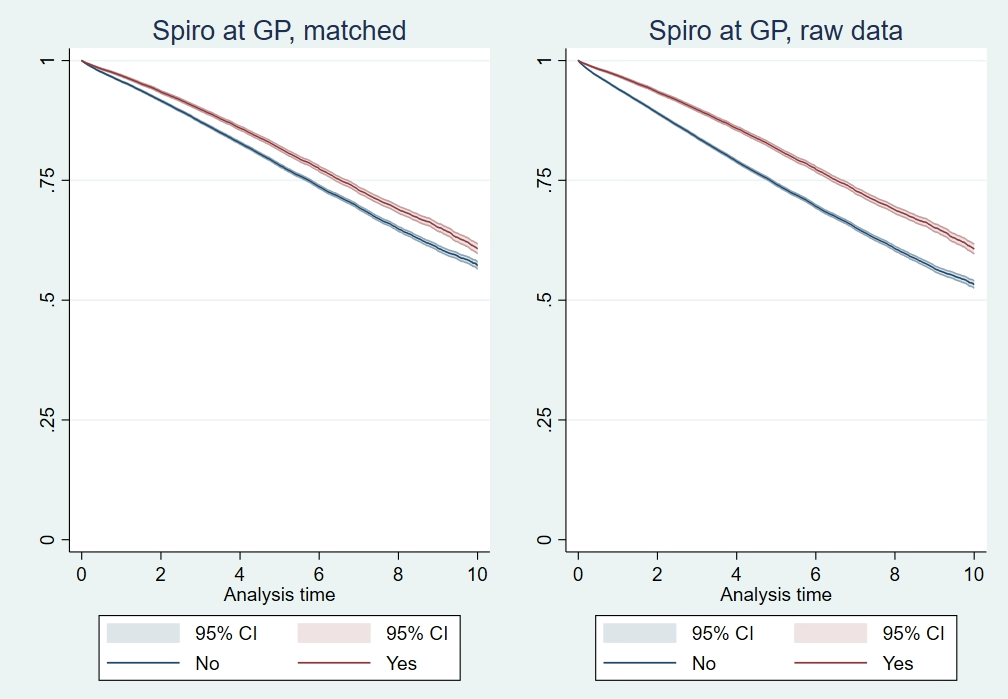


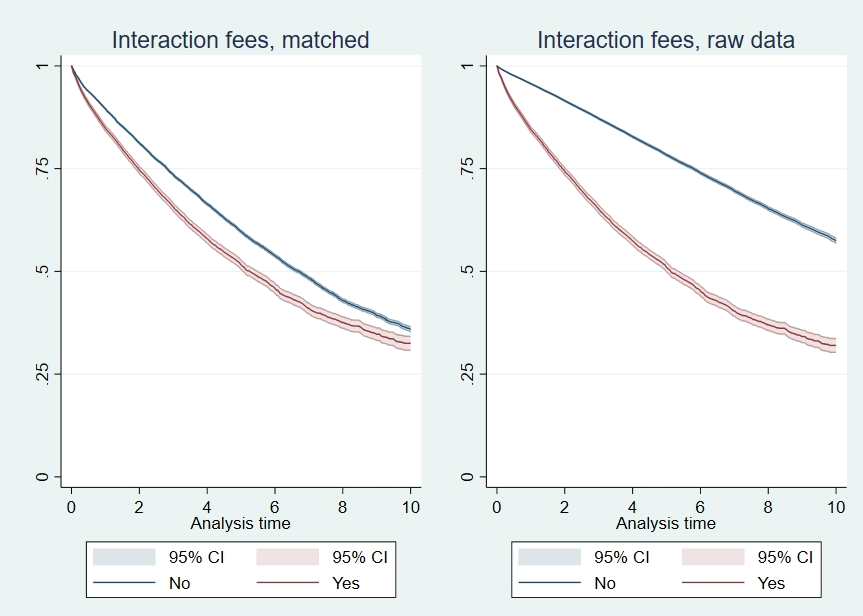


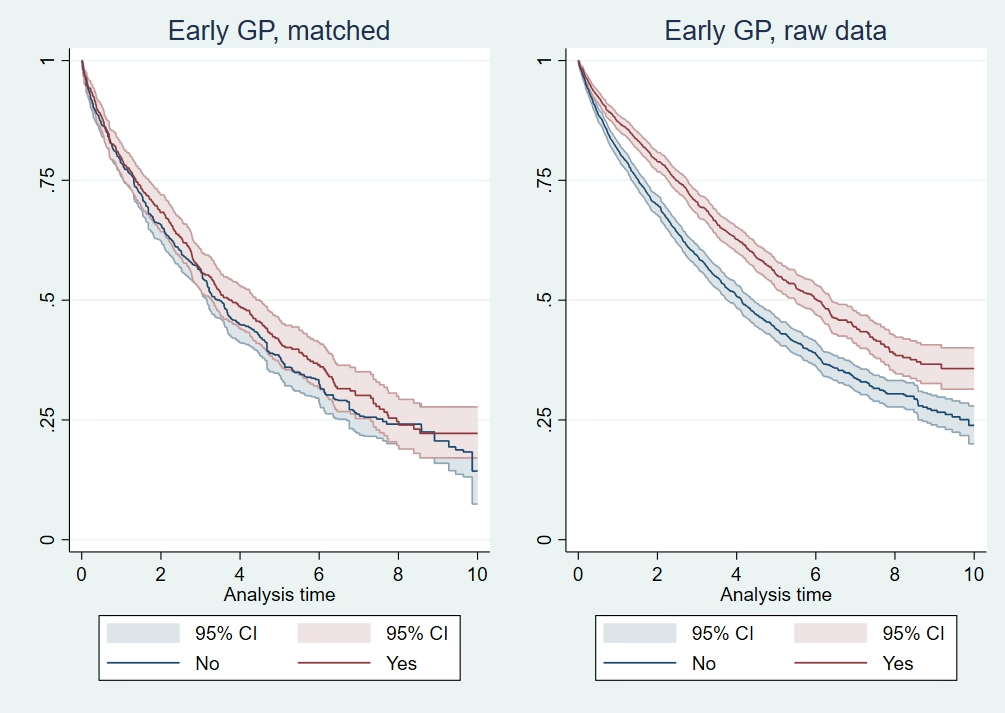


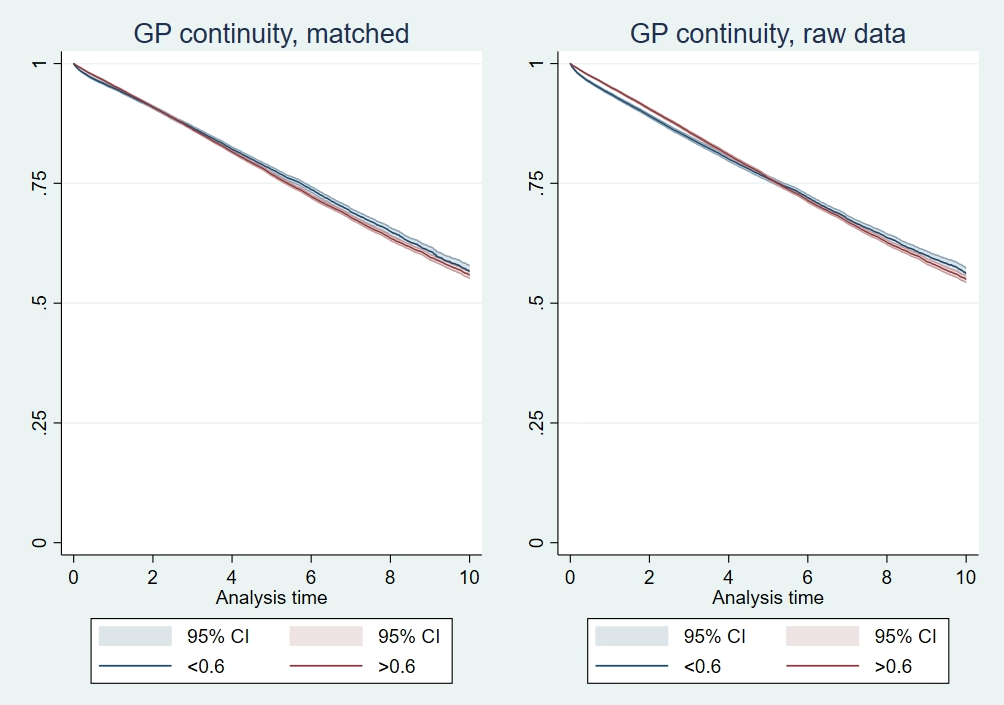


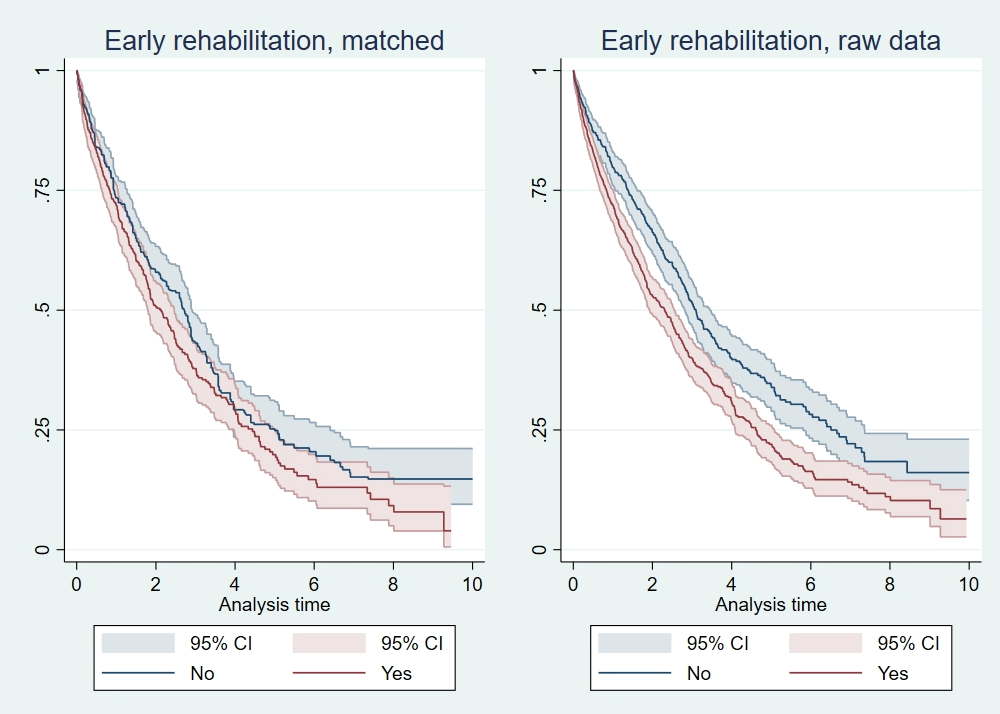


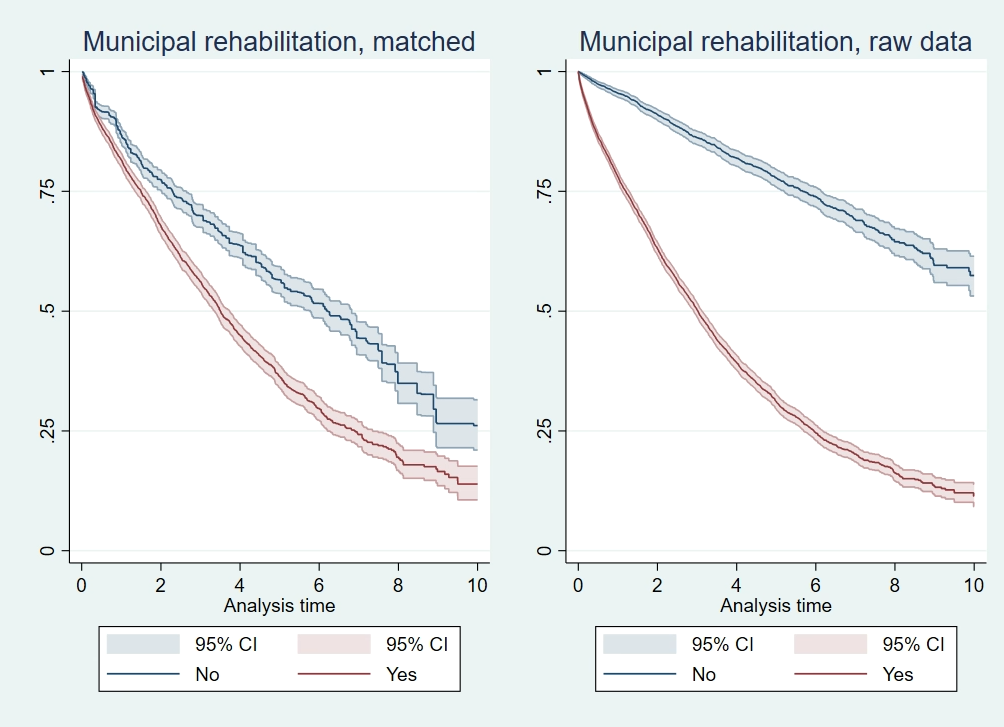


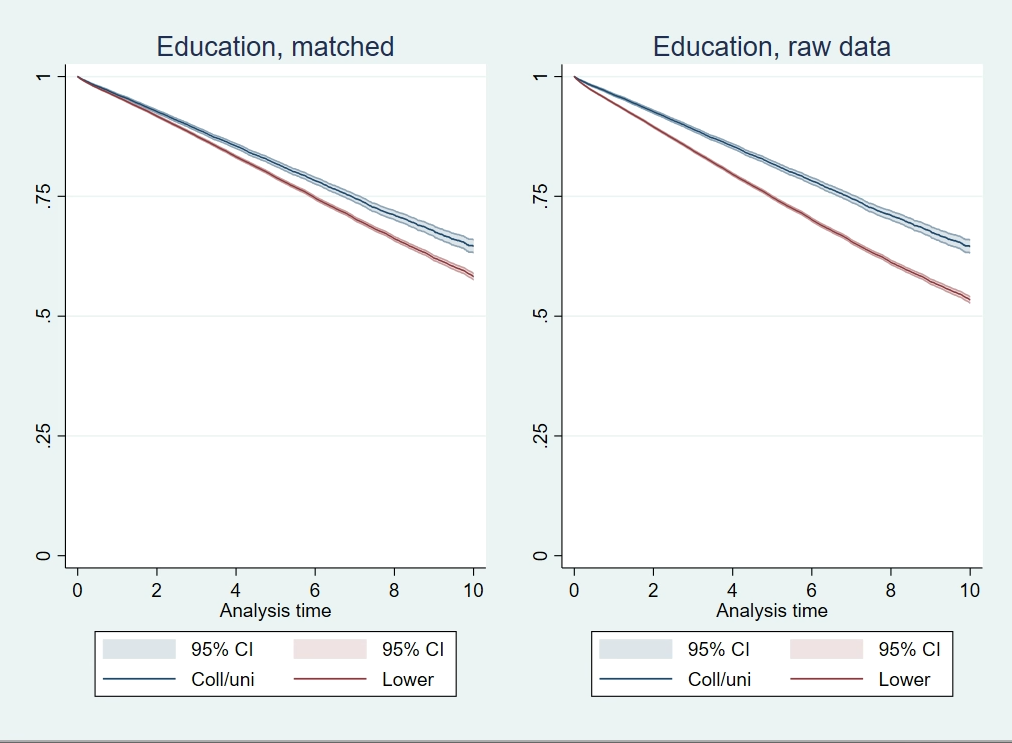


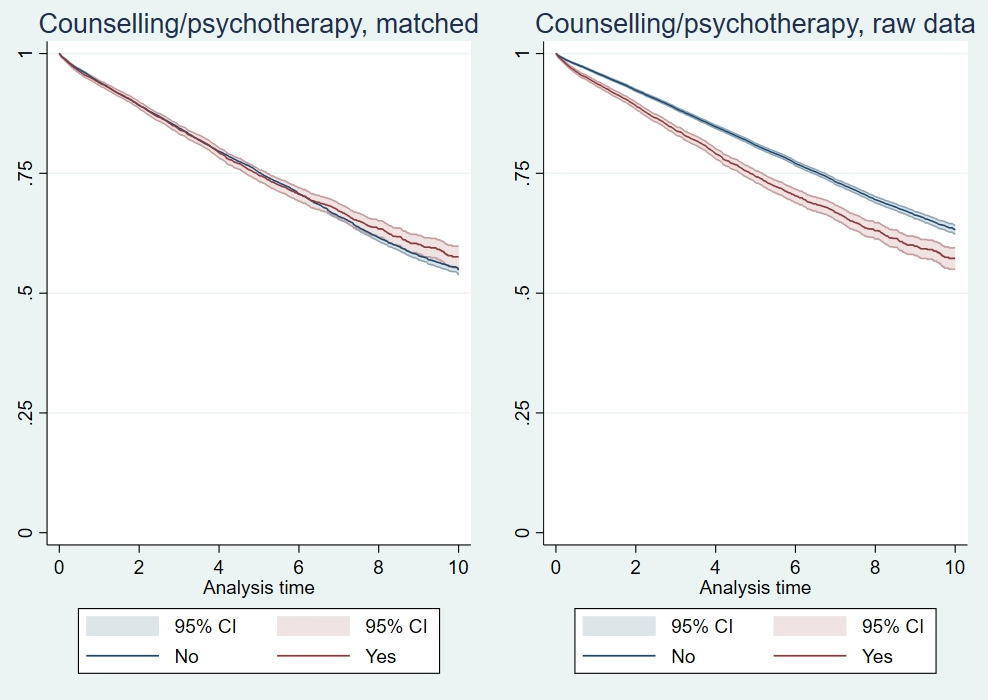

Supplement: Supplementary file 1 — Supplementary Material 1 [file 12913_2026_14285_MOESM1_ESM.docx]
